# Anatomical variations of the marginal mandibular nerve

**DOI:** 10.1016/j.clinsp.2024.100548

**Published:** 2024-12-04

**Authors:** Arthur Paredes Gatti, Matheus Trovão Ribeiro, Flávio Hojaij

**Affiliations:** a Staff of the Head and Neck Surgery Department, Universidade Federal de São Paulo, São Paulo, SP, Brazil; b Hospital das Clínicas da Faculdade de Medicina da Universidade de São Paulo (FMUSPHC), São Paulo, SP, Brazil; c Department of Surgery, Hospital das Clínicas da Faculdade de Medicina da Universidade de São Paulo (FMUSPHC), São Paulo, SP, Brazil

**Keywords:** Anatomy, Mandibular nerve

## Abstract

**Introduction:**

The Marginal Mandibular Nerve (MMN), despite being didactically described as being a unique branch of the other branches of the Facial Nerve, has in practice an abundant anatomical variation. Both in relation to its ramifications and the positioning of these in relation to the lower edge of the mandible, as well as its anastomoses along its path, play a fundamental role in the surgical strategy during the approach of this cervicofacial site, and can generate, as underestimated its characteristics, inadvertent injuries and motor consequences to the patient.

**Objective:**

To analyze the types of anatomical variations of the MMN in relation to its position, ramification, and distance from reference points, as well as its incidence that could imply technical difficulties in surgery.

**Methods:**

A systematic review was conducted in the literature in search of anatomical studies, in vivo or in cadavers, between 1981 and 2024, which described the quantification of the MMN branches, their positioning and peculiarities in relation to anastomoses with other peripheral nerves.

**Results:**

511 hemifaces were studied, with the identification of 290 cases of single nerve, 160 cases of double branch, 54 cases of triple branch, and 7 cases of quadruple branch. Of the 456 nerves that were possible to study, the average distance when superior to the lower limit of the mandible was 1.61 cm, while its lower analysis was 2.53 cm. The branches presented different proportions of presentation in relation to this reference point depending on how many branches it had, being more common to remain below it. Anastomoses were identified between the MMN and the buccal and cervical branch of the Facial Nerve, as well as with the Trigeminal Nerve.

**Conclusion:**

The varied presentations of the MMN, both in its anatomical positioning and in the number of branches leads us to question whether the surgical thinking when performing an exploration in this site may not be the reason whythe authors verify several motor complaints superior to described injuries.

## Highlights

• Most surgeons underestimate the positional and numerical variations of the Facial Nerve.
• About 50% of the Marginal Nerves of the Mandible have more than one subdivision.
• Just over half of the branches of the Mandibular Marginal Nerve are found below the lower border of the mandible.
• The great anatomical variation of the Mandibular Marginal Nerve is responsible for the majority of postoperative motor sequelae.

## Introduction

The Marginal Mandibular Nerve (MMN) originates from the lower trunk (cervicofacial division) of the Facial Nerve (FN). Its main function is related to the motility of facial mimicry, especially the muscles of the mental region and lower lip. Classically,the authors refer to its anatomical position as being related to the lower edge of the parotid gland, running to the lower edge of the mandibular bone and superficially to the posterior facial vein and retromandibular veins in the deep plane of the deep cervical fascia, just below the platysma muscle.^1^^,^^2^ The surgeon's mastery of this anatomy and its variations implies a lower rate of morbidity for the patient.^2^^,^^3^

Despite finding this anatomical description in textbooks,the authors know that the MMN has various anatomical variations, both in terms of its position and the number of branches it has. This variation found both in cadaver studies and observed during surgical procedures that approach this nerve (e.g., parotidectomy and neck dissection), results in a greater difficulty in its location because there is no specific point that can be used in all these anatomical variations.^3^^,^^4^ With this, it is not uncommon for patients undergoing surgeries, mainly in neck dissection, to present after their procedure some degree of facial paralysis in the MMN.^1^, ^2^, ^3^

Both the active search for the MMN and the evaluation of only 1 branch without considering the possibility that it may have more than one can be the causes for this clinical outcome that generates a significant loss of quality of life to the individual, stigmatization, and worse adherence to subsequent treatments.^5^ The surgeon's lack of knowledge of the forms of anatomical variations of the MMN and the difficulty in identifying them in preoperative planning implies greater morbidity for the patient.

In this sense,the authors decided to analyze the types of anatomical variations of the MMN in relation to position, ramifications, and distance from known reference points, in addition to their incidences that could cause technical difficulties in a surgical procedure.

## Materials and methods

A bibliographic review was carried out through PubMed with the descriptors “anatomy” and “mandibular nerve” including articles between 1981 and 2024. Analyses during surgical procedures and cadaver dissection were included. Articles that did not have an individualized analysis of the MMN among the other branches of the FN were excluded. Subsequently, those that had the analysis of the number of subdivisions of MMN branches were separated.

Finally, 8 anatomical studies were separated containing 511 hemifaces with the MMN evaluated in relation to its division, positioning in relation to the lower edge of the mandible and some characteristics of each author in relation to the analysis suggested in their respective works.

## Results

511 hemifaces were studied, including patients intraoperatively and cadavers.^1^^,^^6^, ^7^, ^8^, ^9^, ^10^, ^11^, ^12^ The MMN was evaluated in all cases, either through dissection of the structure *in vivo*, cadaver, or through identification by neurophysiological monitoring of the nerve.

The MMN was identified as a single branch originating from the cervicofacial subdivision or directly from the trunk of the facial nerve in 290 (56.75%) of the cases analyzed. The MMN had 2 branches in 160 (31.31%), 3 branches in 54 (10.57%), and 4 branches in 7 (1.37%).

The distance between the MMN and the lower edge of the mandible could be analyzed in 456 of the 511 studied hemifaces. The average presentation of the MMN superiorly to the lower edge of the mandible was 1.61 cm (3.2‒0.5 cm). The average presentation of the nerve inferiorly to the lower edge of the mandible was 2.53 cm (1.2‒3.6 cm). One of the studies presented an aberrant variation of the MMN and was disregarded due to the possibility of error in relation to the identification of the MMN (9.8–5.0 cm).^1^^,^^6^, ^7^, ^8^, ^9^, ^10^, ^11^, ^12^

Whenthe authors analyzed the predominance of the MMN's position in relation to the lower edge of the mandible,the authors could note that it was located below or on the same line as this reference point in 358 cases (70%) and above it in 153 cases (30%).^1^^,^^6^, ^7^, ^8^, ^9^, ^10^, ^11^, ^12^ Some authors studied the difference between the position of these branches when the MMN had two or more subdivisions. From the two highlighted studies,^6^^,^^9^ the authors were able to analyze 46 MMNs with a total of 109 branches of this nerve. When the MMN had 2 branches (30 MMNs with 60 branches analyzed), 41 (68.3%) were below the lower edge of the mandible, while 19 (31.7%) were above it. Of those that had 3 branches (15 MMNs with 45 branches analyzed), 21 nerves (46.7%) were below the reference point, while 24 (53.3%) were above it. In relation to those that had 4 branches of the MMN (1 patient with 4 branches analyzed), 3 (75%) of their branches were below and 1 (25%) above the lower edge of the mandible.

Some studies evaluated whether these branches of the MMN had any anastomosis during their course. 215 MMNs were analyzed, of which 96 (44.65%) had anastomosis with the buccal branch of the facial nerve, 24 (11.16%) had anastomosis with the cervical branch of the facial nerve, and 14 (6.51%) had anastomosis with the mental nerve, a sensitive branch of the V3 of the trigeminal nerve, along their course.^8^^,^^10^^,^^11^

## Discussion

The FN is composed of approximately 10,000 fibers, predominantly myelinated, whose extracranial function would be to promote the motility of the muscles of facial mimicry.^13^ The path proposed academically is classically presented by 5 unique branches, however, the authors see in practice an abundant anatomical variation. The surgeon, when performing an approach in the higher cervical spaces, for example during neck dissection, when performing the platysma flap, actively searches for the MMN without there being properly an anatomical structure as a parameter but rather a region where it can be identified, leading to the active search for this nerve a more laborious operative step and that can generate neurological injuries, often irreversible. Didactically, the authors have the lower margin of the mandible as the starting point for an imaginary perpendicular measure of 3 cm as the region where the MMN should be located.^14^^,^^15^

By ignoring the fact that this presentation varies, both in relation to the height above or below the lower margin of the mandible and the ramifications and anastomoses that the MMN has, the authors have a high casuistry of neurological changes in this branch, often underdiagnosed or ignored in scientific publications.

This work identified an incidence of just over half of the cases having only 1 branch of the MMN throughout its course (56.75%), showing that almost in the same proportionthe authors have more than one branch that should be identified, preferably those that are below the lower margin of the mandible in the case of a neck dissection surgery. the authors found 2 branches in 31.31% of the cases, followed by 10.57% with 3 branches and 1.37% with 4 branches. These data corroborate with the casuistry in the literature.^16^^,^^17^

Dingman and Grabb^18^ emphasize the importance of the lower edge of the mandible as a point of delimitation of the area to be located in the MMN, as well as the anatomical variation that the presentation of this nerve can have. The survey shows a variation not only in the number of branches but also in what position these branches are in relation to the lower edge of the mandible. In the studies that analyzed this differentiation, the MMNs with 2 branches (60 cases) were completely below the reference point in only 68.3% of the cases, and with 3 branches (15 cases), the majority had a predominance of location above the reference point (53.3%). The only case that was identified 4 branches had 75% of its branches below the reference point.

Farahvash et al.^19^ claim that most of the works underestimate the ramifications of the MMN, even proposing that part of the authors confuse the buccal branch with the marginal in their dissections in cadavers. The present survey identified in the 215 cases analyzed that almost half (44.65%) of the branches had anastomosis between the MMN and the buccal branch of the FN and 6.51% between the MMN and the cervical branch in the FN. In addition to these anastomoses, some authors also refer to anastomosis of the MMN with other nerves, such as the transverse cervical nerve. A study in cadavers identified this anastomosis in all 22 specimens evaluated, with 14 of them this anastomosis occurred near the lower edge of the submandibular gland.^20^ This could explain one of the reasons why the patient, even when the MMN is identified, may present some degree of change in the motility of the corresponding muscles.^21^

Some patients had as a variation of the anastomosis the relationship between the MMN and the trigeminal nerve, which could also justify some complaints of sensitivity change, either in the form of hypoesthesia or facial pain after neck dissection without having occurred manipulation of the trigeminal sensitive innervation.^20^ One of the limitations of this review was precisely the differentiation in some articles of symptoms related specifically to MMN injury and injuries to nerves that have anastomosis between other branches.

The variation according to the distance of the branches in relation to the lower edge of the mandible did not present a great distance from the main studies in the literature, maintaining the idea of a safety limit for the area where the branches tend to be identified up to 3.0 cm below this edge (average of 2.53 cm).^9^^,^^10^^,^^15^ Some authors^22^ presented in a study with 40 cadaver hemifaces, in addition to a high number of cases of multiple branching of the mandibular branch of the facial nerve and anastomoses, a distance of up to 13 mm from the lower edge of the mandible.

In addition to these diverse presentations, the anastomoses that this nerve has along its course near the lower branch of the mandible can affect, in the case of an inadvertent injury, cervicofacial motility and sensitivity. the authors understand that the surgeon's thinking, when approaching this site, keeping the reasoning in identifying a single branch up to 3 cm below the lower edge of the mandible may be the cause of having a number of motor complaints much higher than what is described in the literature as MMN injuries.

Anatomical studies in cadavers and intraoperatively are fundamental for better elucidation of these anatomical variations, which would allow not only a reformulation in the understanding of this nerve but also a change in the didactic concept that affects the intraoperative judgment during dissection for therapeutic purposes.

## Conclusion

There is a great anatomical variation of the MMN in relation to its position in the lower third of the face, in addition to a high incidence of ramifications of this nerve. Although the presentation can vary in relation to the distance of the MMN branches from the lower edge of the mandible, it has a safe distancing interval for its identification. In cases where the surgeon identified multiple branches of the MMN there was less morbidity due to additional intraoperative care with this structure.

The anatomical variation of the MMN tends to be underestimated both by researchers and surgeons, who maintain the didactic idea that this nerve has only 1 branch until its distal termination, which can lead not only to confusion in its identification but also to neurological injuries with unfavorable clinical repercussions on the patient's quality of life.

## Conflicts of interest

The authors declare no conflicts of interest.
